# Genes Versus Lifestyles: Exploring Beliefs About the Determinants of Cognitive Ageing

**DOI:** 10.3389/fpsyg.2022.838323

**Published:** 2022-03-04

**Authors:** Malwina A. Niechcial, Eleftheria Vaportzis, Alan J. Gow

**Affiliations:** ^1^Department of Psychology, Centre for Applied Behavioural Sciences, School of Social Sciences, Heriot-Watt University, Edinburgh, United Kingdom; ^2^Division of Psychology, University of Bradford, Bradford, United Kingdom

**Keywords:** cognitive skills, survey, activities, attribution, brain health

## Abstract

Genetic and lifestyle factors contribute to cognitive ageing. However, the extent to which the public attribute changes in thinking skills to either genetic or lifestyle factors is largely unknown. This may be important if it impacts engagement in activities deemed beneficial to thinking skills. This study, therefore, explored people’s beliefs about determinants of cognitive ageing and whether those beliefs were associated with engagement in potentially beneficial behaviours. Data were collected through a United Kingdom-wide survey of people aged 40 and over. Participants completed questions about their beliefs regarding cognitive ageing, and specifically the extent to which they believed lifestyle or genetic factors influence those changes, and their engagement in specific behaviours that may be cognitively beneficial. Responses from 3,130 individuals (94.0% of the survey sample) were analysed using chi-square tests of independence, principal component analysis and ANCOVAs to investigate whether their attribution of genetic or lifestyle determinants were associated with their beliefs about cognitive ageing and their participation in brain health-related behaviours. Most respondents (62.2%) believed genes and lifestyle contribute equally to age-related changes in cognitive skills. Respondents who believed genetic factors were more influential were less likely to expect cognitive skills might be improved or maintained with age, less sure what behaviours might be associated with brain health, and less likely to engage in behaviours comprising mental challenge/novelty supported as beneficial for brain health. From this United Kingdom-wide survey about beliefs regarding potential determinants of cognitive ageing, some of our respondents’ views were not aligned with the findings from ageing research. It is important for the public to know how to keep their brains healthy. Our results indicate a need for clearer messaging highlighting the role of lifestyle factors for brain health.

## Introduction

Cognitive skills are influenced by our genes and lifestyle. The heritability of general cognitive ability might be as high as 80% during specific periods of the life course (Plomin and Deary, 2015). It may also be that individuals who do well in school and take part in stimulating activities are in fact genetically predisposed to increase their cognitive reserve (Bosma et al., 2002). It has been suggested that different cognitive abilities may have different patterns of heritability with age. For example, Pahlen et al. (2018) suggested genetic influences may decline with age on measures of fluid abilities, such as executive functions and processing speed. For measures of crystallised abilities, such as language skills, genetic influences may increase with age. The authors also suggested that word knowledge may be particularly influenced by gene-environment interactions because it is susceptible to practice, such as reading books (Pahlen et al., 2018). It has previously been estimated that lifestyle factors play a larger role in predicting individual variation in cognitive ageing *trajectories* (Finkel et al., 1998). The first report to use a population-based genetic analysis suggested that about 75% of the variance in cognitive ability from childhood to old age was due to environmental factors (Deary et al., 2012). This can be likened to a specific genetic risk of developing a heart condition, which can be lessened by following a healthy diet and taking up physical exercise. An active and engaged lifestyle is supported as being beneficial for brain health (Fratiglioni et al., 2004; Foresight Mental Capital and Wellbeing Project, 2008; Hertzog et al., 2008; Park et al., 2014), while engaging activities in mid- or late-life may be associated with lower risk of dementia (Valenzuela and Sachdev, 2006; Valenzuela et al., 2011).

Understanding whether people believe genetic or lifestyle factors more strongly determine our thinking skills is important. It is largely unknown whether the public attribute changes in cognitive skills to genetic or lifestyle factors, and how those beliefs impact engagement in behaviours and activities that might be beneficial to those skills. There are three ways knowledge of genetic risks of disease can affect health-related behaviours, namely they can encourage (McClure, 2002), discourage (Hunt et al., 2000) or have no effect on health-related behaviours (Milne et al., 2000). For example, people diagnosed with a familial condition heightening their cholesterol levels were less likely to believe in the contribution of lifestyle factors, instead favouring medication to reduce cholesterol levels (Marteau et al., 2004). If this was similar for cognitive skills (belief in there being predominantly genetic determinants), people would be less likely to believe that brain-healthy behaviours can improve those skills and, therefore, engage in proactive behaviours to a lesser extent. However, those who were informed they were at higher risk of developing Alzheimer’s disease (investigated through APOE ε4 genotyping) were more likely to change their health behaviours (diet, exercise or taking supplements) to reduce their risk than those informed that their genetic risk was not higher (Chao et al., 2008). Those findings may be confounded by there being no effective preventative medication for Alzheimer’s Disease. It is possible, therefore, that given the option of medication people would choose that over lifestyle changes (Marteau et al., 2004). As previous studies have often considered those already more likely to develop a specific condition, it is important to know how people with no prior knowledge of the presence (or absence) of higher risk engage in health-related behaviours.

There are notable differences between younger and older people in their beliefs about changes in cognitive skills. Younger participants are more likely to expect cognitive abilities to decline sooner and more dramatically, which suggests they may be guided by stereotypes about ageing. That is in contrast to older adults who are more likely to expect those abilities to be better maintained or show a more gradual loss. This could be the product of lived experience, either their own and/or those of their family and friends (Hertzog and Hultsch, 2000; Vaportzis and Gow, 2018). Previously reported findings from the current survey suggested older respondents had more optimistic expectations about when cognitive changes might begin, that is, that changes might begin later (Vaportzis and Gow, 2018). Our intention was, therefore, to extend those analyses and consider whether age differences in people’s beliefs about cognitive ageing might also be reflected in their attributions of genetic versus lifestyle determinants of those changes.

It may be that general beliefs around ageing (also cognitive) may differ from beliefs individuals have about themselves. For example, although older adults may be more optimistic when estimating the timing of expected (or experienced) changes in cognitive skills on average (Hertzog and Hultsch, 2000; Vaportzis and Gow, 2018), they have been found to be more likely to evaluate their cognitive abilities as poorer and report more problems with their memory than younger adults (Zanardo et al., 2006). Additionally, older adults (especially those over the age of 75) were more likely than younger adults to attribute greater importance to uncontrollable factors in terms of age-related changes (Zanardo et al., 2006). For example, observing health-related declines may prompt older adults to feel less in control (Robinson and Lachman, 2017). This might suggest that those over 75 expect their cognitive abilities are dictated by factors they cannot control (such as, genes) and may, therefore, perceive cognitive ageing as fixed.

Attitudes toward ageing, especially with regard to the malleability or fixedness of ageing on a person’s traits and attributes (which can include intelligence), and the influence of such attitudes on task performance has been considered (Plaks and Chasteen, 2013; Neel and Lassetter, 2015; Weiss et al., 2016; Weiss, 2018). While this evidence presents a compelling case for predicting peoples’ beliefs about cognitive ageing and engagement in brain-healthy behaviours, brain health is rarely the focus of such surveys, which led to the development of the current approach.

Phelan and colleagues conducted a survey to explore what people believe is important for successful ageing (Phelan et al., 2004). Having the kind of genes to age successfully was important to over 60% of respondents and being able to make lifestyle choices affecting how we age was important to over 85% of respondents (Phelan et al., 2004). Surveys on brain health in the United Kingdom and United States found that more than 9 in 10 respondents believe cognitive skills can be improved with age (AARP, 2015; Vaportzis and Gow, 2018). While knowledge of potentially beneficial lifestyle factors varied, those rated as most important to maintaining or improving cognitive skills from the United Kingdom survey included having a sense of purpose, eating a healthy diet, challenging the mind, getting adequate sleep, and keeping physically active (Vaportzis and Gow, 2018). Although people recommended many of those lifestyles and behaviours to others to improve or maintain their cognitive skills with age (Niechcial et al., 2019) and reported engaging in many of them, they rarely did so to specifically benefit their cognitive skills (AARP, 2015; Vaportzis and Gow, 2018).

Females may also be more likely than males to believe people can learn new things at any age, and more willing and motivated to improve their cognitive skills compared to males (AARP, 2014, 2017; Vaportzis and Gow, 2018). On the other hand, older people reported being less willing to do something to improve their cognitive skills (AARP, 2017). Younger people are more likely to believe their thinking skills may be improved. Understanding the varying “windows of opportunity” to introduce positive brain health messaging at different stages of the lifespan may, therefore, be possible but requires a better understanding of what people are likely to believe during different life stages. Taking that knowledge forward then, middle-aged adults (aged 40–65) may be able to introduce new habits in good time to improve their cognition (Gates et al., 2019). It is important to understand their beliefs about cognitive ageing to maximise the potential of health messaging focused on lifestyle changes to help them make proactive choices sooner. Ultimately, this may mean that these interventions implemented in ample time have the desired effect of reducing or eliminating the risk of cognitive decline and dementia (Gates et al., 2019). Attributions of how changes in cognitive skills might be determined by genetic and lifestyle factors, and the engagement in lifestyles and behaviours deemed beneficial to those skills, were, therefore, examined in a large, representative survey of adults aged 40 and over in the United Kingdom.

### Objectives

This study was conducted to answer the following questions:

• What do people believe is the balance of genetic and lifestyle factors as determinants of age-related changes in cognitive skills?
• Is self-reported cognitive health associated with attribution of genetic/lifestyle determinants?
• Do people’s beliefs about the possibility of improving/maintaining cognitive skills with age vary according to their attribution of genetic/lifestyle determinants?
• Do the behaviours people report doing vary according to their attribution of genetic/lifestyle determinants?

Although this survey was exploratory, based on previous literature we expected that respondents who believe that lifestyle factors are the more important determinant of cognitive ageing will report better cognitive health and be more likely to believe their cognitive skills can be improved/maintained. We also predicted that respondents endorsing lifestyle factors as the dominant determinant of cognitive ageing would be more likely to report participating in activities that are associated with improved brain health. Finally, we expected younger respondents to be more likely to endorse lifestyle factors as the more important determinant of cognitive ageing.

## Materials and Methods

### Participants

“What Keeps You Sharp?” was a United Kingdom-wide survey conducted between November 2016 and February 2017. Respondents were 3,330 adults aged 40 and over. They completed the survey online or in hardcopy. The survey explored their attitudes to and beliefs about how cognitive skills might change with age (Vaportzis and Gow, 2018). Online data collection was facilitated through a market research company, Survey Sampling (*N* = 2,327). The online survey was additionally circulated via social groups, General Practitioner (personal doctor) practices, our volunteer database and contacts with Age UK and Age Scotland (*N* = 883). Our team distributed hardcopy surveys and the second author (E.V.) visited community centres to directly recruit the oldest-old and those without internet access (*N* = 120). Fuller details of the survey methodology are available (Vaportzis and Gow, 2018).

Of the 3,330 responses, 113 participants missed the current questions of interest, giving a completion rate of 96.6% (*N* = 3,217). The intended sample was adults aged 40 and over, therefore, 27 respondents who reported to be younger than 40 were excluded. Responses of those not reporting their age were also excluded (*N* = 60). The analytical sample was, therefore, 3,130 (94.0% of the overall respondents), comprising 1,837 females (58.7%), 1,286 males (41.1%), with five respondents identifying as “other” (0.2%) and two not reporting their gender (0.1%). Most respondents identified as white British (91.5%, *N* = 2,863). The mean age was 59.9 years old (*SD* = 11.1, range 40-98). For age comparisons, we created three groups: “middle-aged” comprising 40–59 year olds (*N* = 1,503, 48.0%), “young-old” comprising those aged 60–79 (*N* = 1,529, 48.8%) and “old-old” 80–98 year olds (*N* = 98, 3.1%). Our respondents were relatively well educated with 71.6% (*N* = 2,241) having completed either college, professional qualifications, undergraduate or postgraduate degrees. More specifically, 27.2% of our respondents (*N* = 850) completed lower education (including primary and secondary school), 36.5% (*N* = 1,144) completed middle education (including college and professional training), 35.0% (*N* = 1,097) completed higher education (including undergraduate and postgraduate degrees), and 1.2% (*N* = 39) completed other education. Responses may not total 3,130 because of missing data.

The project was approved by the Heriot-Watt University School of Social Sciences Ethics Committee (ref 2016/340). All respondents gave informed consent either in writing or by agreeing to four consent questions included at the start of the online survey.

### Survey

The survey contained questions related to cognitive ageing, including when respondents thought different cognitive skills declined and whether they believed cognitive skills can be maintained or improved with age (reported previously, Vaportzis and Gow, 2018). The current paper focuses on respondents’ beliefs regarding the genetic and lifestyle contributors to age-related changes in cognitive skills, how those beliefs were associated with behaviours respondents were involved in, and whether they did those behaviours because they believed they were good for their cognitive skills.

Within the larger survey we asked our respondents the following questions relevant to the current analyses:

“Our lifestyles sometimes affect how healthy we are as we age. Some health outcomes might also be influenced by our genes. I think that the changes we experience in our thinking skills as we age are (choose what you think may be most likely)” to which respondents could choose “entirely determined by our genes,” “mostly determined by our genes,” “probably about half determined by our genes and half determined by our lifestyle,” “mostly determined by our lifestyle” or “entirely determined by our lifestyle”;

“Do you think there are things people can do to maintain or improve their thinking skills as they grow older?” responses to which were “yes,” “no” or “not sure”;

“How would you rate your thinking skills or mental sharpness?” where they could indicate their skills were “excellent,” “very good,” “good,” “fair” or “poor”;

“Do you know how to keep your brain healthy?” to which they could respond “yes,” “no” or “not sure”; and

“Below is a list of activities that some people believe might be good for their thinking skills. We want you to tell us which of the following activities you regularly do and which you do because they might be good for your thinking skills. If you do not do the activity, select “Don’t do,”” where they could respond “don’t do,” “regularly do” or “do because it is good for your thinking skills.” The list consisted of 19 lifestyles and behaviours, such as learning new things, eating a healthy diet, pursuing a purpose in life, volunteering and helping others, and managing stress effectively (Supplementary Table 1).

### Data Analysis

To investigate how respondents’ beliefs regarding the genetic/lifestyle determinants of cognitive ageing were associated with their cognitive health and views about improvement of cognitive skills with age, the categorical data were put into contingency tables and analysed by chi-square (χ*^2^*) tests of independence. For *post hoc* tests, we squared the adjusted standardised residual scores produced to convert them into χ*^2^* values for each comparison. We then estimated *p*-values from those by computing a new variable using the χ*^2^* significance function in SPSS and compared those individual *p*-values against Bonferroni corrected *p*-value for each comparison (Beasley and Schumacker, 1995; MacDonald and Gardner, 2000). This method was also used in our previous report on the survey (Vaportzis and Gow, 2018).

We also investigated the associations between respondents’ beliefs regarding determinants of cognitive ageing and their engagement in the 19 behaviours. The three response options were collapsed to two by combining “regularly do” and “do because it is good for your thinking skills” (i.e., becoming “do” and “don’t do”). We conducted a Principal Component Analysis to identify the underlying factor structure from the 19 behaviours. In brief, the overall Kaiser-Meyer-Olkin (KMO) measure of sampling adequacy (MSA) was 0.81 and no individual item MSA was lower than 0.66. The scree plot suggested five factors (accounting for 45.5% of the overall variance), which were extracted by direct oblimin rotation. Factor loadings are given in Supplementary Table 1, defining Purposeful activity; Health behaviours; Games; Mindful and creative activity; Informational activity.

The five factors were used in ANCOVAs investigating associations between respondents’ beliefs regarding determinants of cognitive ageing and the behaviours they were involved in, with age group and gender as covariates.

## Results

### What Do People Believe Is the Balance of Genetic and Lifestyle Factors as Determinants of Age-Related Changes in Cognitive Skills?

Overall, 62.2% of respondents believed that cognitive skills are determined equally by genes and lifestyle. There was a significant association between the attribution of genetic/lifestyle determinants and respondents’ gender (*p* < 0.05, Table 1). Although males and females mostly believed in an equal influence of genetic/lifestyle determinants on cognitive skills, *post hoc* tests revealed that females (66.5%) were significantly more likely than males (56.3%) to believe in the equal influence; males were significantly more likely than females to indicate cognitive skills are mostly (18.0% males vs. 11.2% females) or entirely (4.1% males vs. 2.4% females) determined by genes.

**TABLE 1:** Respondents’ opinions about determinants of cognitive skills broken down by age group and gender.

Characteristics	Cognitive skills are entirely determined by genes	Cognitive skills are mostly determined by genes	Cognitive skills are determined equally by genes and lifestyle	Cognitive skills are mostly determined by lifestyle	Cognitive skills are entirely determined by lifestyle	χ*^2^*
Total *n* (%)	96 (3.1)	438 (14.1)	1,937 (62.2)	582 (18.7)	59 (1.9)	
**Age group**						
Middle-aged *n* (%)	51 (3.4)	224 (14.9)	937 (62.3)	264 (17.6)	27 (1.8)	χ^2^(8) = 11.47
Young-old *n* (%)	42 (2.8)	197 (13.0)	956 (62.9)	296 (19.5)	29 (1.9)	*p* = 0.177
Old-old *n* (%)	3 (3.4)	17 (19.1)	44 (49.4)	22 (24.7)	3 (3.4)	*N* = 3,112
**Gender**						
Female *n* (%)	43 (2.4)^a^	205 (11.2)^a^	1,214 (66.5)^a^	329 (18.0)	34 (1.9)	χ^2^(4) = 46.48
Male *n* (%)	53 (4.1)^a^	231 (18.0)^a^	720 (56.3)^a^	252 (19.7)	24 (1.9)	*p* < 0.001* *N* = 3,105

Overall χ^2^ are significant at *p < 0.05.

*^a^Denotes post hoc comparisons significant at the p < 0.0056 level.*

Although respondents in the old-old group (aged 80–98) were less likely to believe in this equal influence (49.4% compared with 62.3% of the middle-aged group and 62.9% of the young-old group), these age differences were not significant (*p* > 0.05, Table 1). The old-old were also more likely to choose “mostly determined by our genes” (19.1% compared with 14.9% of the middle-aged group and 13.0% of the young-old group) or “mostly determined by our lifestyle” (24.7% compared with 17.6% of the middle-aged group and 19.5% of the young-old group), however these differences were also not significant (*p* > 0.05, Table 1).

For the remaining analyses, we pooled responses of “entirely determined by genes” and “mostly determined by genes” into a single “genes” category; similarly, a “lifestyle” category combined “entirely determined by lifestyle” and “mostly determined by lifestyle” responses. The “probably about half determined by genes and half determined by lifestyle” category will be referred to as “equal.” This resulted in three categories, “genes,” “equal” and “lifestyle.”

### Is Self-Reported Cognitive Health Associated With Attribution of Genetic/Lifestyle Determinants?

The χ*^2^* test of independence suggested no association between self-rated cognitive ability and whether people believed cognitive skills were determined by genes or lifestyle (*p* > 0.05, Table 2).

**TABLE 2:** Chi-square tests examining respondents’ beliefs about cognitive skills according to their opinions about determinants of those skills.

Beliefs about cognitive skills	Genes *n* (%)	Equal *n* (%)	Lifestyle *n* (%)	χ*^2^*
**My cognitive skills are**				
Excellent	90 (19.1)	271 (57.7)	109 (23.2)	χ*^2^* (8) = 14.09
Very good	256 (17.1)	926 (61.8)	316 (21.1)	*p* = 0.080
Good	154 (17.2)	568 (63.6)	171 (19.1)	*N* = 3,109
Fair	27 (12.4)	155 (71.1)	36 (16.5)	
Poor	6 (20.0)	16 (53.3)	8 (26.7)	
**Cognitive skills can be maintained/improved with age**				
Yes	453 (15.9)^a^	1,783 (62.7)	609 (21.4)^a^	χ^2^(4) = 69.63
Not sure	41 (22.7)	118 (65.2)	22 (12.2)^a^	*p* < 0.001*
No	40 (48.2)^a^	34 (41.0)^a^	9 (10.8)	*N* = 3,109
**I know how to keep my brain healthy**				
Yes	285 (15.6)^a^	1,131 (61.8)	413 (22.6)^a^	χ^2^(4) = 36.19
Not sure	167 (16.9)	643 (64.9)	181 (18.3)	*p* < 0.001*
No	82 (17.2)^a^	161 (62.2)	46 (20.6)	*N* = 3,109

Overall χ^2^ are significant at *p < 0.05.

*^a^Denotes post hoc comparisons significant at the p < 0.0056 level.*

### Do People’s Beliefs About the Possibility of Improving/Maintaining Cognitive Skills With Age Vary According to Their Attribution of Genetic/Lifestyle Determinants?

There was a significant association between the attribution of genetic/lifestyle determinants and people’s beliefs about whether cognitive skills could be improved/maintained with age (*p* < 0.05, Table 2). *Post hoc* tests revealed that those who believed genes made a greater contribution to changes in cognitive skills were less likely to think those skills could be maintained/improved with age; those who believed lifestyle made a greater contribution to changes in cognitive skills as well as those who believed in equal attribution of both determinants were more likely to think skills could be maintained/improved with age (Table 2).

There was also a significant association between the attribution of genetic/lifestyle determinants and respondents’ certainty of knowing what might be good for brain health (*p* < 0.05, Table 2). *Post hoc* tests revealed that those who believed genes made a greater contribution were less likely to know what might help improve cognitive skills, while those who believed lifestyle made a greater contribution were more likely to report knowing how to maintain/improve cognitive skills (Table 2).

### Do the Behaviours People Report Doing Vary According to Their Attribution of Genetic/Lifestyle Determinants?

**FIGURE 1:**
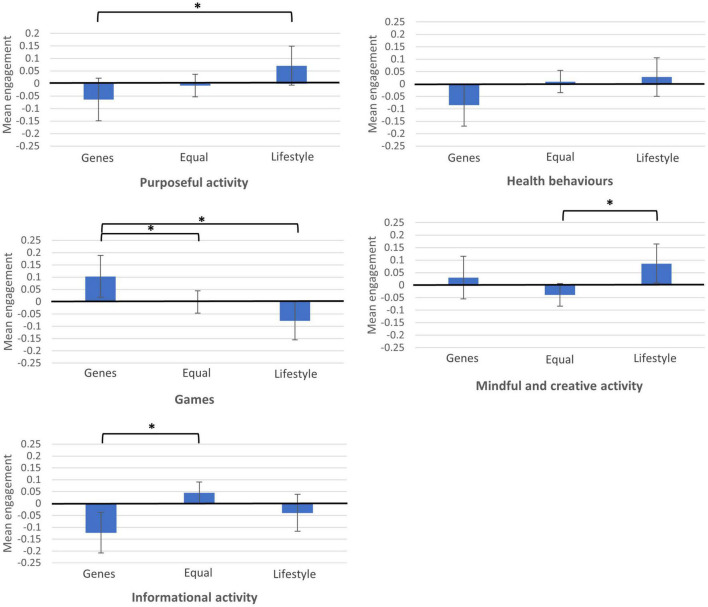
Mean differences in engagement in five behaviours by determinants of cognitive skills. *Mean differences are significant at *p* < 0.05.

We carried out five separate ANCOVAs to explore the associations between people’s attributions of genetic/lifestyle determinants of cognitive skills and the five factors we identified from behaviours they reported doing, with age and gender as covariates (Table 3 and Figure 1). The attribution of determinants was significantly associated with Games (*F* = 4.776, *p* < 0.01, η*p*^2^ = 0.003), Mindful and creative activity (*F* = 4.171, *p* < 0.05, η*p*^2^ = 0.003), and Informational activity (*F* = 6.369, *p* < 0.01, η*p*^2^ = 0.004). The overall results were, therefore, followed-up with pairwise comparisons.

**TABLE 3:** ANCOVAs examining respondents’ engagement in behaviours according to their opinions about determinants of cognitive skills.

Behaviours engaged in by attribution of determinants of cognitive skills	*F*	*p*	η*_*p*_*^2^	Mean difference	*p*
**Purposeful activity**	2.746	0.064	0.002		
Genes—equal				–0.056	0.260
Equal—lifestyle				–0.079	0.083
Lifestyle—genes				0.135	0.022*
**Health behaviours**	2.271	0.103	0.001		
Genes—equal				–0.095	0.052
Equal—lifestyle				–0.018	0.698
Lifestyle—genes				0.113	0.053
**Games**	4.776	0.008*	0.003		
Genes—equal				0.103	0.036*
Equal—lifestyle				0.078	0.088
Lifestyle—genes				–0.181	0.002*
**Mindful and creative activity**	4.171	0.016*	0.003		
Genes—equal				0.069	0.159
Equal—lifestyle				–0.126	0.005*
Lifestyle—genes				0.057	0.328
**Informational activity**	6.369	0.002*	0.004		
Genes—equal				–0.167	0.001*
Equal—lifestyle				0.083	0.067
Lifestyle—genes				0.084	0.152

Analyses of covariance for each factor and mean differences are significant at *p < 0.05.

The *post hoc* comparisons suggested people who attributed changes in cognitive skills to genes had higher Games scores vs. all others (mean difference 0.103, *p* < 0.05 between genes and equal, and mean difference 0.181, *p* < 0.05 between genes and lifestyle). Those attributing changes to lifestyle had higher Mindful and creative activity scores vs. those attributing equal genes/lifestyle (mean difference 0.126, *p* < 0.05). Finally, those believing in a genetic attribution had lower Informational activity scores than those believing in equal attribution (mean difference −0.167, *p* < 0.05). In summary, people who believed that genes more strongly influence cognitive ageing were more likely to engage in games and brain training, and less likely to watch news and educational programmes.

There were no significant differences between beliefs about determinants of cognitive skills and Purposeful activity, and Health behaviours (*p* > 0.05) overall, although there was a significant difference specifically between genes and lifestyle in Purposeful activity (mean difference −0.135, *p* < 0.05). Those who believed in a greater contribution of genes to cognitive ageing were less likely to engage in behaviours, such as learning new things and taking part in educational classes.

## Discussion

This was the first United Kingdom-wide survey asking adults about their beliefs regarding the genetic and lifestyle determinants of cognitive ageing. Most of our respondents believed that changes in cognitive skills were equally determined by genetic and lifestyle factors, which is perhaps less optimistic than empirical evidence that lifestyle plays a larger role (Deary et al., 2012). In our sample, while females and males mostly believed in the equal influence of genes and lifestyle, females were more likely than males to report this belief; males were more likely than females to believe that genes influence our cognitive skills to a greater extent. Females were previously reported to be more likely than males to believe people can learn new things at any age, and more willing and motivated to improve their cognitive skills compared to males (AARP, 2014, 2017; Vaportzis and Gow, 2018). We might, therefore, suggest that is partly due to a higher proportion of males believing in a stronger influence of genetic factors, potentially perceiving those abilities to be less malleable.

There were no significant differences between the three age groups in their beliefs about the genetic/lifestyle contributions, though this could be due to a very small number of respondents in the old-old group. This is somewhat in contrast with the literature on differences across age groups in perceptions of age-related cognitive changes (Hertzog and Hultsch, 2000). Previous studies have shown that older adults (and especially those over 75) are more likely to report uncontrollable factors as more important with regards to age-related changes (Zanardo et al., 2006; Robinson and Lachman, 2017). By extension, we might expect older adults to report less malleable and more rigid views of the determinants of cognitive ageing (i.e., a stronger endorsement of genetic factors). Although to some extent these differences were exhibited by our respondents, the small size of the old-old group relative to other groups limited our ability to make those comparisons, warranting further exploration.

Self-ratings of cognitive ability were not associated with beliefs about the contributions of genes and lifestyle to changes in cognitive skills. However, those beliefs were associated with how malleable they thought cognitive skills might be in later life and whether they knew how to support those changes. Those beliefs were also associated with the behaviours our respondents reported doing. Respondents believing that genes were the stronger influence were more likely to engage in games and brain training and less likely to read, watch the news and engage in educational programmes.

It could be argued that those who think genetic factors are more important to cognitive ageing are engaging more in behaviours with unclear benefits for brain health, such as brain training, and less in behaviours deemed good for cognitive skills, comprising mental challenge and novelty, such as learning new things or participating in educational activities. This might provide partial support to studies linking genetic risks for some diseases and health-related behaviours (Marteau et al., 2004; Chao et al., 2008), where the individuals at risk show preference for ways that might appear more direct in terms of preventing the disease (in this case endorsing brain training) than a potentially less direct/more challenging lifestyle change, such as trying new activities. While respondents who believed genetic factors were the stronger contributor still engaged in certain behaviours, they were not more likely to engage in mentally stimulating activities, such as novel learning, which has been reported as an important contributor to maintaining/improving cognitive skills (Park et al., 2014). On the other hand, respondents who believed in an equal influence of genetic and lifestyle factors were less likely to engage in behaviours, such as meditation and playing a musical instrument. This is somewhat in line with previous research (AARP, 2015; Vaportzis and Gow, 2018) suggesting that people generally do not engage in certain behaviours, although they would recommend those behaviours to others to improve their cognitive skills (Niechcial et al., 2019). This finding points to a need for brain health messaging highlighting the importance of engaging in brain-healthy activities and behaviours.

Our survey was carried out in the United Kingdom and although our findings are consistent with US-derived data, beliefs and attitudes toward cognitive ageing may differ in other countries and cultures. Future surveys should focus on non-Western countries to allow comparisons; the multi-country, multilanguage Global Brain Health Survey is a positive development in that regard (Budin-Ljosne et al., 2020). Our study does not allow in-depth analysis of the data reported. Future studies should seek to explore the context-rich aspects of people’s reported beliefs, perhaps supplementing the survey methodology with qualitative designs. Equally, as this was a cross-sectional survey, we cannot imply that beliefs determine behaviour; studies supporting behaviour change via modifying beliefs and understanding about brain health would be of value. Our survey was exploratory in nature and examined the relationship of people’s beliefs regarding genetic and lifestyle factors contributing to their cognitive skills in later life and their engagement in potentially brain-healthy behaviours. We did not set out to test any particular theory in our survey, which can be seen as a limitation of our work. In light of previous literature, such as malleability and fixedness of ageing, and capacity for change (Plaks and Chasteen, 2013; Neel and Lassetter, 2015; Weiss et al., 2016; Weiss, 2018), we would only be able to offer speculative suggestions about our findings.

Although descriptive, our results suggest that a higher proportion of males than females believe that cognitive skills are influenced by our genes rather than lifestyle factors, which may mean they see those skills as less malleable. People who believe genes are the greater contributor may not believe cognitive skills can be improved with age, appear less likely to know how to improve those skills, and consequently may not engage in activities and behaviours that could benefit those skills. Our results indicate a need for clear brain health messaging to ensure the general public’s beliefs are in line with current empirical findings and that everyone knows how to maintain their cognitive skills with age. This could be achieved through resources, such as Age UK’s Staying Sharp webpages (Age UK, 2017) and AARP’s online resources related to brain health (AARP, n.d.). It is also important that this messaging reaches people at an earlier age (for example, from midlife), so that they may take proactive steps toward brain health throughout the life course. It would be useful, for example, to highlight that lifestyle factors may have a potentially greater role in improving or maintaining brain health, especially given those factors are more likely to be amenable to personal control.

## Data Availability Statement

The data, analytic methods, and study materials relating to this study will be made available to other researchers upon request. This survey was not preregistered.

## Ethics Statement

The studies involving human participants were reviewed and approved by Heriot-Watt University School of Social Sciences Ethics Committee. The patients/participants provided their written informed consent to participate in this study.

## Author Contributions

AJG and EV designed the study and collected the data. MAN and AJG analysed the data. MAN drafted the manuscript. All authors made substantial contributions, revised this manuscript, and approved the final version to be published.

## Conflict of Interest

The authors declare that the research was conducted in the absence of any commercial or financial relationships that could be construed as a potential conflict of interest.

## Publisher’s Note

All claims expressed in this article are solely those of the authors and do not necessarily represent those of their affiliated organizations, or those of the publisher, the editors and the reviewers. Any product that may be evaluated in this article, or claim that may be made by its manufacturer, is not guaranteed or endorsed by the publisher.
