# Supplementary material for: Genes Versus Lifestyles: Exploring Beliefs About the Determinants of Cognitive Ageing
Source: Front Psychol. 2022 Mar 4;13:838323. doi: 10.3389/fpsyg.2022.838323 (PMC8931720; doi:10.3389/fpsyg.2022.838323)
Supplement: Supplementary file 1 [file Table_1.DOCX]

**S1 Table** Item loadings from Principal Component Analysis

| Variable | Component | | | | |
| --- | --- | --- | --- | --- | --- |
|  | 1 | 2 | 3 | 4 | 5 |
| Trying new activities | 0.796 | 0.163 | 0.119 | 0.217 | 0.173 |
| Learning new things | 0.783 | 0.181 | 0.129 | 0.070 | 0.208 |
| Pursuing hobbies | 0.518 | 0.337 | 0.238 | 0.033 | 0.205 |
| Taking educational classes | 0.507 | 0.027 | -0.048 | 0.507 | 0.121 |
| Having a purpose in life | 0.489 | 0.471 | 0.014 | 0.128 | 0.252 |
| Volunteering or helping others | 0.441 | 0.190 | 0.001 | 0.376 | 0.203 |
| Getting enough sleep | 0.105 | 0.707 | 0.045 | 0.077 | 0.056 |
| Managing stress effectively | 0.154 | 0.695 | 0.197 | 0.002 | 0.027 |
| Eating a healthy diet | 0.128 | 0.508 | -0.058 | 0.260 | 0.352 |
| Socialising with friends or family | 0.292 | 0.466 | 0.014 | -0.019 | 0.327 |
| Exercising your body or physical activity | 0.249 | 0.430 | -0.093 | 0.330 | 0.298 |
| Challenging the mind with games,  puzzles, or other activities | 0.154 | 0.102 | 0.787 | -0.017 | 0.144 |
| Playing games designed for brain training | 0.105 | 0.085 | 0.773 | 0.247 | 0.057 |
| Engaging in mindful activities such as meditation, yoga or prayer | 0.255 | 0.110 | 0.139 | 0.717 | 0.099 |
| Playing a musical instrument or singing | 0.258 | 0.061 | 0.095 | 0.601 | 0.063 |
| Taking vitamins or supplements | -0.109 | 0.066 | 0.012 | 0.522 | 0.040 |
| Watching the news | -0.038 | 0.114 | -0.004 | -0.061 | 0.726 |
| Watching educational programmes | 0.255 | 0.034 | 0.162 | 0.224 | 0.633 |
| Reading | 0.229 | 0.178 | 0.039 | 0.053 | 0.498 |

*Note.* Items loading over ~0.5 were referred to in naming the factors, however, all items contributed to each factor score according to their respective loadings. The factors were defined as Factor 1: Purposeful activity; Factor 2: Health behaviours; Factor 3: Games; Factor 4: Mindful and creative activity; Factor 5: Informational activity.
